# Exploring the effects of theta burst stimulation on craving severity and biomarkers in patients with gambling disorder

**DOI:** 10.1192/j.eurpsy.2025.635

**Published:** 2025-08-26

**Authors:** H.-M. Chang

**Affiliations:** 1 Institute of Epidemiology and Preventive Medicine, National Taiwan University; 2 Songde Branch, Taipei City Hospital. Taipei, Taiwan, Province of China

## Abstract

**Introduction:**

Gambling disorder, previously known as pathological gambling, is a behavior that significantly impairs functioning in personal, social, and occupational domains. Currently, there is no pharmacological treatment for gambling disorder, emphasizing the need for innovative treatment modalities. Imaging studies have identified a connection between prefrontal circuit dysfunction and behavioral disinhibition, supporting the potential use of non-invasive brain stimulation in treating gambling disorder.

**Objectives:**

The purpose of this study is to investigate the effect of theta-burst stimulation (TBS) on gambling disorder.

**Methods:**

The study duration is 2 weeks, with 10 sessions of TBS intervention. The intervention group will receive 1800 pulses of intermittent TBS at the left dorsolateral prefrontal cortex and 1200 pulses of continuous TBS at the pre-supplementary motor area during each session, while the control group will receive sham stimulation. Primary outcomes, including the Gambling Symptom Assessment Scale (G-SAS) and the Visual Analogue Scale (VAS) for craving, were administered at weeks 0, 2, 4, and 8, and the changes between the two groups were compared using generalized estimating equations. Secondary outcomes, including Beck Anxiety Inventory and Beck Depression Inventory, and serum brain-derived neurotrophic factor (BDNF), cortisol, and hsCRP, were measured at weeks 0, 4, and the changes between the two groups were compared using repeated measures ANOVA.

**Results:**

A total of 33 patients with gambling disorder were randomly assigned in a 2:1 ratio to the intervention group (21 patients) and the control group (12 patients) on a double-blind basis. They were included in the preliminary analysis on an intention-to-treat basis. The VAS scores of the active group decreased more than those of the sham group (active group: 53.3 to 17.9, sham group: 37.5 to 15.3), but the difference did not reach statistical significance (*p* = 0.13). Compared to the sham group, the active group showed a decreasing trend in hsCRP (*p* = 0.54) and an increasing trend in free BDNF (*p* = 0.34), but neither reached a statistically significant difference.

**Conclusions:**

Drawing definitive conclusions is limited by small sample size. Nevertheless, the initial results from this study suggest that the alterations in levels of gambling craving, hsCRP, and serum BDNF align with our hypothesis.

**Disclosure of Interest:**

None Declared

